# Ethyl 2-allylsulfanyl-4-(4-methoxyphenyl)-6-methyl-1,4-dihydropyrimidine-5-carboxylate

**DOI:** 10.1107/S1600536808026664

**Published:** 2008-08-23

**Authors:** M. Nizam Mohideen, A. Rasheeth, C. A. M. A. Huq

**Affiliations:** a Department of Physics, The New College (Autonomous), Chennai 600 014, India; b Department of Chemistry, The New College (Autonomous), Chennai 600 014, India

## Abstract

In the title compound, C_18_H_22_N_2_O_3_S, the pyrimidine ring is not planar. It adopts a half-chair conformation The crystal structure is characterized by classical N—H⋯O and C—H⋯O inter- and intra­molecular hydrogen bonds, respectively. The title compound exhibits a wide spectrum of biological activities.

## Related literature

For related literature, see: Allen *et al.* (1987 ▶); Biginelli (1893 ▶); Cremer & Pople (1975 ▶); Gurskaya *et al.* (2003*a* ▶,*b* ▶); Kappe (1993 ▶); Kappe *et al.* (1997 ▶); Li (2006 ▶); Nardelli (1983 ▶); Nizam Mohideen *et al.* (2008 ▶); Overman *et al.* (1995 ▶); Snider *et al.* (1996 ▶).
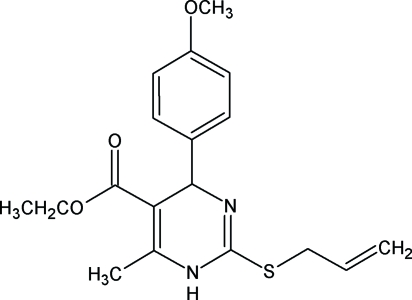


## Experimental

### 

#### Crystal data

C_18_H_22_N_2_O_3_S
*M* *_r_* = 346.44
Monoclinic, 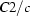

*a* = 28.325 (5) Å
*b* = 7.410 (2) Å
*c* = 20.202 (4) Å
β = 121.61 (3)°
*V* = 3610.9 (18) Å^3^
*Z* = 8
Mo *K*α radiation
μ = 0.20 mm^−1^
*T* = 293 (2) K
0.4 × 0.2 × 0.1 mm

#### Data collection

Bruker Kappa APEXII CCD diffractometer
Absorption correction: multi-scan (*SADABS*; Bruker, 2004 ▶) *T*_min_ = 0.954, *T*_max_ = 0.983
16675 measured reflections
3183 independent reflections
2722 reflections with *I* > 2σ(*I*)
*R*_int_ = 0.025

#### Refinement

*R*[*F* ^2^ > 2σ(*F* ^2^)] = 0.045
*wR*(*F* ^2^) = 0.140
*S* = 1.04
3183 reflections
220 parameters
H-atom parameters constrained
Δρ_max_ = 0.47 e Å^−3^
Δρ_min_ = −0.33 e Å^−3^

### 

Data collection: *APEX2* (Bruker, 2004 ▶); cell refinement: *APEX2* and *SAINT* (Bruker, 2004 ▶); data reduction: *SAINT* and *XPREP* (Bruker, 2004 ▶); program(s) used to solve structure: *SHELXS97* (Sheldrick, 2008 ▶); program(s) used to refine structure: *SHELXL97* (Sheldrick, 2008 ▶); molecular graphics: *ORTEP-3 for Windows* (Farrugia, 1997 ▶); software used to prepare material for publication: *SHELXL97* and *PLATON* (Spek, 2003 ▶).

## Supplementary Material

Crystal structure: contains datablocks global, I. DOI: 10.1107/S1600536808026664/pv2100sup1.cif

Structure factors: contains datablocks I. DOI: 10.1107/S1600536808026664/pv2100Isup2.hkl

Additional supplementary materials:  crystallographic information; 3D view; checkCIF report

## Figures and Tables

**Table 1:** Hydrogen-bond geometry (Å, °)

*D*—H⋯*A*	*D*—H	H⋯*A*	*D*⋯*A*	*D*—H⋯*A*
N2—H2⋯O2^i^	0.86	2.16	2.990 (2)	161
C7—H7⋯O2	0.98	2.46	2.831 (3)	102

Symmetry code: (i) 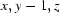.

**Fig. 1.**
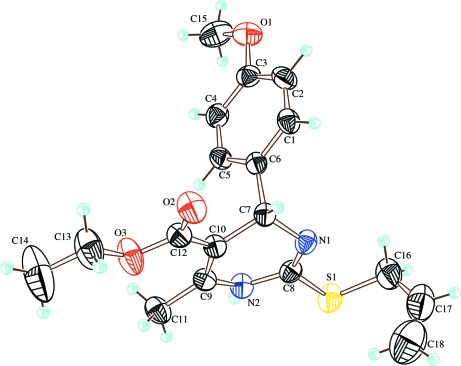
The molecular configuration and atom-numbering scheme for (I). Displacement ellipsoids are drawn at the 50% probability level.

## supplementary crystallographic information

### Comment

The title compound, (I), belongs to the class of 5-substituted 1,2,3,4-tetrahydropyrimidin-2-ones, which are known as `Biginelli compounds' (Kappe, 1993). The Biginelli reaction is a classic multicomponent reaction (Biginelli, 1893). The biological activity of some isolated alkaloids has been attributed to the presence of the dihydropyrimidinone moiety in the molecules (Overman *et al.*, 1995) and the conformation of the pyrimidine ring (Kappe *et al.*, 1997; Gurskaya *et al.*, 2003*a*,b). Most important among them are batzelladine alkaloids, which have been found to be potent HIVgp-120-CD4 inhibitors (Snider *et al.*, 1996). The aim of the present work was to study classical and extended Biginelli reactions. As part of our ongoing investigation of pyrimidine derivatives, the title compound, (I), has been prepared and its crystal structure is presented here.

The bond lengths and angles in the title compound (Fig. 1) are comparable with ethyl 1,2,3,4-tetrahydro-6-methyl-2-oxo-4-phenylpyrimidine-5-carboxylate, a structure closely related to (I) (Nizam Mohideen *et al.*, 2008). The torsion angles [C1—C6—C7—C10 = 153.1 (2), C5—C6—C7—C10 = -31.5 (2), C9—C10—C12—O2 = 171.3 (2), C7—C10—C12—O2 = -11.6 (3), C9—C10—C12—O3 = -10.1 (1) and C7—C10—C12—O3 = 167.1 (2) °] differs from the torsion angles [47.6 (2), -137.1 (2), 10.1 (2), -167.8 (2) -171.5 (2) and 10.5 (2) °] in the reported structure mentioned above.

In (I), the heterocyclic ring (atoms N1, N2, C7, C8, C9, C10) of the dihydropyrimidine group is not planar, as indicated by the displacement of atom C7 from the least-squares plane [0.212 (1) Å] and by the C8—N1—C7—C10 torsion angle [31.1 (1) °]. Atom C11 deviating by -0.204 (1) Å from the least squares plane of the pyrimidine ring. The pyrimidine ring adopts half chair conformation; the puckering parameters are q_2_ = 0.312 (1) Å, φ = 236.3 (2)°, and θ = 104.2 (1)° (Cremer & Pople, 1975), and the lowest displacement asymmetry parameters Δ_S_(C7) is 2.3 (1)°, Δ_2_(C10) is 22.4 (1)° (Nardelli, 1983).

The benzene ring is planar, the larget displacement observed being -0.008 (1) Å for atom C6. The dihedral angle between the pyrimidine and benzene rings is 89.5 (1)°, close to the value of 86.5 (1)° found in ethyl 1,2,3,4-tetrahydro-6-methyl-2-oxo-4-phenylpyrimidine-5-carboxylate.

The crystal packing is characterized by classical N—H···O and C—H···O inter and intramolecular hydrogen bonds (Table 1).

### Experimental

To a suspension of NaH (0.100 g, 2 mmol, 50% dispersion in mineral oil washed with hexane) in dry THF (25 ml) was added a solution of dihydropyrimidone, (0.594 g, 2 mmol) in dry THF (10 ml) and stirred in an atmosphere of N_2_ for one hour. Then a solution of allyl bromide (0.2 ml, 2.5 mmol) in dry THF (5 ml) was added drop wise and stirred for futher four hours. (TLC control, silica, ethyl acetate: hexane 1:9 as eluent). Evaporation of solvent under reduced pressure, followed by purification of the residue by column chromatography gave a yellow solid. Single crystals of the title compound suitable for X-ray diffraction were obtained by slow evaporation of a solution in ethanol (mp 368–369 K).

### Refinement

All H atoms were positioned geometrically and allowed to ride on their parent C atoms, with C—H distances fixed in the range 0.93–0.98 Å and N—H distance of 0.86 Å, with *U*_iso_(H) = 1.5*U*_eq_(C) for methyl H atoms and *U*_iso_(H) = 1.2*U*_eq_(C, N) for other H atoms.

### Crystal data

**Table:**

C_18_H_22_N_2_O_3_S	*F*_000_ = 1472
*M**_r_* = 346.44	*D*_x_ = 1.275 Mg m^−^^3^
Monoclinic, *C*2/*c*	Mo *K*α radiation λ = 0.71073 Å
Hall symbol: -C 2yc	Cell parameters from 7889 reflections
*a* = 28.325 (5) Å	θ = 2.6–25º
*b* = 7.410 (2) Å	µ = 0.20 mm^−^^1^
*c* = 20.202 (4) Å	*T* = 293 (2) K
β = 121.61 (3)º	Needle, yellow
*V* = 3610.9 (18) Å^3^	0.4 × 0.2 × 0.1 mm
*Z* = 8	

### Data collection

**Table:**

Bruker Kappa APEXII CCD diffractometer'	3183 independent reflections
Radiation source: fine-focus sealed tube	2722 reflections with *I* > 2σ(*I*)
Monochromator: graphite	*R*_int_ = 0.026
*T* = 293(2) K	θ_max_ = 25.0º
ω and φ scan	θ_min_ = 1.7º
Absorption correction: Multi-scan(SADABS; Bruker, 2004)	*h* = −33→33
*T*_min_ = 0.954, *T*_max_ = 0.983	*k* = −8→8
16675 measured reflections	*l* = −24→24

### Refinement

**Table:**

Refinement on *F*^2^	Secondary atom site location: difference Fourier map
Least-squares matrix: full	Hydrogen site location: inferred from neighbouring sites
*R*[*F*^2^ > 2σ(*F*^2^)] = 0.045	H-atom parameters constrained
*wR*(*F*^2^) = 0.140	*w* = 1/[σ^2^(*F*_o_^2^) + (0.0785*P*)^2^ + 3.5811*P*] where *P* = (*F*_o_^2^ + 2*F*_c_^2^)/3
*S* = 1.05	(Δ/σ)_max_ = 0.002
3183 reflections	Δρ_max_ = 0.48 e Å^−^^3^
220 parameters	Δρ_min_ = −0.33 e Å^−^^3^
Primary atom site location: structure-invariant direct methods	Extinction correction: none

### Special details

**Table:**

Geometry. All e.s.d.'s (except the e.s.d. in the dihedral angle between two l.s. planes) are estimated using the full covariance matrix. The cell e.s.d.'s are taken into account individually in the estimation of e.s.d.'s in distances, angles and torsion angles; correlations between e.s.d.'s in cell parameters are only used when they are defined by crystal symmetry. An approximate (isotropic) treatment of cell e.s.d.'s is used for estimating e.s.d.'s involving l.s. planes.
Refinement. Refinement of *F*^2^ against ALL reflections. The weighted *R*-factor *wR* and goodness of fit *S* are based on *F*^2^, conventional *R*-factors *R* are based on *F*, with *F* set to zero for negative *F*^2^. The threshold expression of *F*^2^ > σ(*F*^2^) is used only for calculating *R*-factors(gt) *etc*. and is not relevant to the choice of reflections for refinement. *R*-factors based on *F*^2^ are statistically about twice as large as those based on *F*, and *R*- factors based on ALL data will be even larger.

### Fractional atomic coordinates and isotropic or equivalent isotropic displacement parameters (Å^2^)

**Table:**

	*x*	*y*	*z*	*U*_iso_*/*U*_eq_	
S1	0.15041 (3)	0.63300 (8)	0.44134 (3)	0.0551 (2)	
O1	0.33272 (7)	1.1779 (2)	0.34204 (10)	0.0580 (4)	
O2	0.06407 (7)	1.4020 (2)	0.24877 (9)	0.0522 (4)	
O3	0.01426 (7)	1.2100 (2)	0.15153 (9)	0.0617 (5)	
N1	0.14728 (7)	0.9848 (2)	0.40787 (9)	0.0385 (4)	
N2	0.09759 (7)	0.7816 (2)	0.30377 (10)	0.0387 (4)	
H2	0.0954	0.6715	0.2889	0.046*	
C1	0.23063 (8)	1.2512 (3)	0.39532 (11)	0.0399 (5)	
H1	0.2280	1.3171	0.4325	0.048*	
C2	0.27875 (9)	1.2591 (3)	0.39437 (13)	0.0456 (5)	
H2A	0.3082	1.3292	0.4308	0.055*	
C3	0.28341 (8)	1.1622 (3)	0.33898 (12)	0.0406 (5)	
C4	0.23922 (9)	1.0596 (3)	0.28488 (12)	0.0424 (5)	
H4	0.2418	0.9951	0.2474	0.051*	
C5	0.19079 (8)	1.0533 (3)	0.28672 (11)	0.0387 (5)	
H5	0.1611	0.9844	0.2499	0.046*	
C6	0.18558 (8)	1.1467 (2)	0.34195 (11)	0.0331 (4)	
C7	0.13489 (8)	1.1274 (2)	0.34918 (11)	0.0334 (4)	
H7	0.1298	1.2415	0.3692	0.040*	
C8	0.13171 (8)	0.8265 (3)	0.38169 (11)	0.0359 (4)	
C9	0.06696 (8)	0.9154 (3)	0.25004 (11)	0.0355 (4)	
C10	0.08227 (8)	1.0897 (3)	0.27177 (11)	0.0341 (4)	
C11	0.02055 (9)	0.8432 (3)	0.17415 (13)	0.0484 (5)	
H11A	−0.0117	0.9173	0.1562	0.073*	
H11B	0.0123	0.7217	0.1813	0.073*	
H11C	0.0315	0.8444	0.1365	0.073*	
C12	0.05336 (8)	1.2485 (3)	0.22475 (12)	0.0382 (5)	
C13	−0.01773 (13)	1.3590 (4)	0.10104 (16)	0.0718 (8)	
H13A	0.0067	1.4473	0.0989	0.086*	
H13B	−0.0387	1.4178	0.1204	0.086*	
C14	−0.05525 (19)	1.2832 (6)	0.0238 (2)	0.1255 (18)	
H14A	−0.0341	1.2191	0.0066	0.188*	
H14B	−0.0755	1.3789	−0.0120	0.188*	
H14C	−0.0807	1.2017	0.0261	0.188*	
C15	0.33997 (12)	1.0703 (4)	0.28976 (19)	0.0700 (8)	
H15A	0.3327	0.9462	0.2949	0.105*	
H15B	0.3774	1.0823	0.3016	0.105*	
H15C	0.3147	1.1098	0.2374	0.105*	
C16	0.19334 (12)	0.7289 (4)	0.53535 (15)	0.0662 (7)	
H16A	0.2215	0.6410	0.5676	0.079*	
H16B	0.2122	0.8324	0.5304	0.079*	
C17	0.16579 (19)	0.7873 (5)	0.57661 (19)	0.0867 (10)	
H17	0.1891	0.8174	0.6287	0.104*	
C18	0.1139 (2)	0.8017 (6)	0.5498 (3)	0.1070 (13)	
H18A	0.0884	0.7736	0.4981	0.128*	
H18B	0.1015	0.8404	0.5820	0.128*	

### Atomic displacement parameters (Å^2^)

**Table:**

	*U*^11^	*U*^22^	*U*^33^	*U*^12^	*U*^13^	*U*^23^
S1	0.0785 (5)	0.0335 (3)	0.0492 (4)	−0.0010 (3)	0.0305 (3)	0.0058 (2)
O1	0.0468 (9)	0.0614 (11)	0.0707 (11)	−0.0059 (8)	0.0342 (8)	−0.0012 (9)
O2	0.0615 (10)	0.0249 (8)	0.0522 (9)	0.0007 (7)	0.0174 (8)	−0.0004 (7)
O3	0.0692 (11)	0.0348 (9)	0.0446 (9)	0.0068 (8)	0.0045 (8)	0.0012 (7)
N1	0.0498 (10)	0.0318 (9)	0.0347 (9)	−0.0012 (7)	0.0226 (8)	0.0015 (7)
N2	0.0510 (10)	0.0219 (8)	0.0409 (9)	0.0009 (7)	0.0224 (8)	−0.0030 (7)
C1	0.0456 (11)	0.0325 (11)	0.0356 (10)	−0.0020 (8)	0.0171 (9)	−0.0050 (8)
C2	0.0414 (11)	0.0391 (12)	0.0444 (11)	−0.0100 (9)	0.0142 (9)	−0.0054 (9)
C3	0.0393 (11)	0.0361 (11)	0.0454 (11)	0.0010 (8)	0.0215 (9)	0.0083 (9)
C4	0.0521 (12)	0.0385 (11)	0.0400 (11)	−0.0019 (9)	0.0264 (10)	−0.0029 (9)
C5	0.0419 (11)	0.0337 (11)	0.0358 (10)	−0.0082 (8)	0.0171 (9)	−0.0065 (8)
C6	0.0395 (10)	0.0237 (9)	0.0313 (9)	0.0004 (7)	0.0151 (8)	0.0028 (7)
C7	0.0415 (10)	0.0240 (9)	0.0331 (9)	−0.0002 (8)	0.0184 (8)	−0.0013 (7)
C8	0.0436 (11)	0.0294 (10)	0.0383 (10)	0.0022 (8)	0.0239 (9)	0.0033 (8)
C9	0.0372 (10)	0.0307 (10)	0.0387 (10)	−0.0005 (8)	0.0200 (9)	−0.0024 (8)
C10	0.0376 (10)	0.0270 (10)	0.0359 (10)	0.0006 (8)	0.0180 (8)	−0.0002 (8)
C11	0.0488 (13)	0.0345 (12)	0.0484 (12)	−0.0045 (9)	0.0161 (10)	−0.0061 (9)
C12	0.0380 (10)	0.0333 (11)	0.0405 (11)	0.0007 (8)	0.0187 (9)	0.0004 (8)
C13	0.0752 (18)	0.0447 (15)	0.0550 (15)	0.0156 (13)	0.0063 (13)	0.0100 (12)
C14	0.133 (3)	0.085 (3)	0.065 (2)	0.022 (2)	−0.013 (2)	0.0010 (19)
C15	0.0728 (17)	0.0696 (18)	0.094 (2)	0.0004 (14)	0.0614 (17)	0.0047 (16)
C16	0.0784 (18)	0.0486 (15)	0.0485 (14)	−0.0047 (13)	0.0173 (13)	0.0134 (11)
C17	0.129 (3)	0.067 (2)	0.0587 (17)	−0.014 (2)	0.045 (2)	−0.0015 (15)
C18	0.141 (4)	0.098 (3)	0.103 (3)	0.017 (3)	0.078 (3)	0.004 (2)

### Geometric parameters (Å, °)

**Table:**

S1—C8	1.766 (2)	C7—H7	0.9800
S1—C16	1.780 (3)	C9—C10	1.359 (3)
O1—C3	1.370 (3)	C9—C11	1.501 (3)
O1—C15	1.421 (3)	C10—C12	1.464 (3)
O2—C12	1.211 (2)	C11—H11A	0.9600
O3—C12	1.334 (3)	C11—H11B	0.9600
O3—C13	1.453 (3)	C11—H11C	0.9600
N1—C8	1.267 (3)	C12—O2	1.211 (2)
N1—C7	1.486 (2)	C13—C14	1.464 (4)
N2—C8	1.388 (3)	C13—H13A	0.9700
N2—C9	1.389 (3)	C13—H13B	0.9700
N2—H2	0.8600	C14—H14A	0.9600
C1—C2	1.374 (3)	C14—H14B	0.9600
C1—C6	1.395 (3)	C14—H14C	0.9600
C1—H1	0.9300	C15—H15A	0.9600
C2—C3	1.393 (3)	C15—H15B	0.9600
C2—H2A	0.9300	C15—H15C	0.9600
C3—C4	1.380 (3)	C16—C17	1.474 (5)
C4—C5	1.392 (3)	C16—H16A	0.9700
C4—H4	0.9300	C16—H16B	0.9700
C5—C6	1.385 (3)	C17—C18	1.277 (5)
C5—H5	0.9300	C17—H17	0.9300
C6—C7	1.524 (3)	C18—H18A	0.9300
C7—C10	1.517 (3)	C18—H18B	0.9300
			
C8—S1—C16	101.35 (11)	C9—C11—H11B	109.5
C3—O1—C15	117.37 (19)	H11A—C11—H11B	109.5
C12—O3—C13	117.77 (18)	C9—C11—H11C	109.5
C8—N1—C7	116.11 (16)	H11A—C11—H11C	109.5
C8—N2—C9	119.55 (16)	H11B—C11—H11C	109.5
C8—N2—H2	120.2	O2—C12—O3	122.22 (18)
C9—N2—H2	120.2	O2—C12—O3	122.22 (18)
C2—C1—C6	121.64 (19)	O2—C12—C10	123.91 (19)
C2—C1—H1	119.2	O2—C12—C10	123.91 (19)
C6—C1—H1	119.2	O3—C12—C10	113.86 (17)
C1—C2—C3	120.11 (19)	O3—C13—C14	107.1 (2)
C1—C2—H2A	119.9	O3—C13—H13A	110.3
C3—C2—H2A	119.9	C14—C13—H13A	110.3
O1—C3—C4	124.2 (2)	O3—C13—H13B	110.3
O1—C3—C2	116.34 (19)	C14—C13—H13B	110.3
C4—C3—C2	119.44 (19)	H13A—C13—H13B	108.5
C3—C4—C5	119.65 (19)	C13—C14—H14A	109.5
C3—C4—H4	120.2	C13—C14—H14B	109.5
C5—C4—H4	120.2	H14A—C14—H14B	109.5
C6—C5—C4	121.81 (18)	C13—C14—H14C	109.5
C6—C5—H5	119.1	H14A—C14—H14C	109.5
C4—C5—H5	119.1	H14B—C14—H14C	109.5
C5—C6—C1	117.34 (18)	O1—C15—H15A	109.5
C5—C6—C7	122.25 (17)	O1—C15—H15B	109.5
C1—C6—C7	120.26 (17)	H15A—C15—H15B	109.5
N1—C7—C10	112.63 (15)	O1—C15—H15C	109.5
N1—C7—C6	107.63 (15)	H15A—C15—H15C	109.5
C10—C7—C6	112.61 (15)	H15B—C15—H15C	109.5
N1—C7—H7	107.9	C17—C16—S1	116.9 (2)
C10—C7—H7	107.9	C17—C16—H16A	108.1
C6—C7—H7	107.9	S1—C16—H16A	108.1
N1—C8—N2	125.34 (17)	C17—C16—H16B	108.1
N1—C8—S1	123.53 (15)	S1—C16—H16B	108.1
N2—C8—S1	111.13 (14)	H16A—C16—H16B	107.3
C10—C9—N2	117.60 (18)	C18—C17—C16	128.1 (3)
C10—C9—C11	128.98 (19)	C18—C17—H17	115.9
N2—C9—C11	113.43 (17)	C16—C17—H17	115.9
C9—C10—C12	125.37 (18)	C17—C18—H18A	120.0
C9—C10—C7	118.86 (17)	C17—C18—H18B	120.0
C12—C10—C7	115.70 (16)	H18A—C18—H18B	120.0
C9—C11—H11A	109.5		
			
C6—C1—C2—C3	0.3 (3)	C16—S1—C8—N2	179.62 (16)
C15—O1—C3—C4	−5.1 (3)	C8—N2—C9—C10	17.0 (3)
C15—O1—C3—C2	175.6 (2)	C8—N2—C9—C11	−163.18 (18)
C1—C2—C3—O1	179.86 (19)	N2—C9—C10—C12	−176.65 (17)
C1—C2—C3—C4	0.5 (3)	C11—C9—C10—C12	3.6 (3)
O1—C3—C4—C5	−179.79 (19)	N2—C9—C10—C7	6.3 (3)
C2—C3—C4—C5	−0.5 (3)	C11—C9—C10—C7	−173.39 (19)
C3—C4—C5—C6	−0.4 (3)	N1—C7—C10—C9	−29.8 (2)
C4—C5—C6—C1	1.2 (3)	C6—C7—C10—C9	92.1 (2)
C4—C5—C6—C7	−174.30 (18)	N1—C7—C10—C12	152.88 (16)
C2—C1—C6—C5	−1.2 (3)	C6—C7—C10—C12	−85.2 (2)
C2—C1—C6—C7	174.42 (18)	C13—O3—C12—O2	−2.9 (3)
C8—N1—C7—C10	31.2 (2)	C13—O3—C12—O2	−2.9 (3)
C8—N1—C7—C6	−93.6 (2)	C13—O3—C12—C10	178.3 (2)
C5—C6—C7—N1	93.2 (2)	C9—C10—C12—O2	171.3 (2)
C1—C6—C7—N1	−82.1 (2)	C7—C10—C12—O2	−11.6 (3)
C5—C6—C7—C10	−31.5 (2)	C9—C10—C12—O2	171.3 (2)
C1—C6—C7—C10	153.14 (17)	C7—C10—C12—O2	−11.6 (3)
C7—N1—C8—N2	−10.0 (3)	C9—C10—C12—O3	−10.0 (3)
C7—N1—C8—S1	170.98 (14)	C7—C10—C12—O3	167.10 (17)
C9—N2—C8—N1	−16.0 (3)	C12—O3—C13—C14	177.0 (3)
C9—N2—C8—S1	163.17 (14)	C8—S1—C16—C17	90.0 (2)
C16—S1—C8—N1	−1.2 (2)	S1—C16—C17—C18	−10.8 (5)

### Hydrogen-bond geometry (Å, °)

**Table:**

*D*—H···*A*	*D*—H	H···*A*	*D*···*A*	*D*—H···*A*
N2—H2···O2^i^	0.86	2.16	2.990 (2)	161
C7—H7···O2	0.98	2.46	2.831 (3)	102

Symmetry codes: (i) *x*, *y*−1, *z*.
